# Retraction: Effect of zinc nanoparticles seed priming and foliar application on the growth and physio-biochemical indices of spinach (*Spinacia oleracea* L.) under salt stress

**DOI:** 10.1371/journal.pone.0272431

**Published:** 2022-08-17

**Authors:** The PLOS ONE Editors

The *PLOS ONE* Editors retract this article [1] because it was identified as one of a series of submissions for which we have concerns about authorship, competing interests, and peer review. We regret that the issues were not addressed prior to the article’s publication.

SZ, MKK, MRS, RH, NS, AAS, TJ, and MHS did not agree with the retraction. SP, SR, MN, GF, SA, and MI either did not respond directly or could not be reached.
